# Recent advances in understanding and combatting *Mycoplasma genitalium*

**DOI:** 10.12703/b/9-3

**Published:** 2020-10-30

**Authors:** Joanne Peel, Ei Aung, Stephanie Bond, Catriona Bradshaw

**Affiliations:** 1 Melbourne Sexual Health Centre, Alfred Health, Melbourne, VIC, Australia; 2 Central Clinical School, Faculty of Medicine, Nursing and Health Sciences, Monash University, Melbourne, VIC, Australia

**Keywords:** Mycoplasma genitalium, anti-microbial resistance

## Abstract

*Mycoplasma genitalium* has emerged over the last 30 years as a sexually transmitted infection (STI). As data have accumulated, our understanding of this pathogen and its role in disease continues to evolve. This in turn creates new challenges and complexities. Questions remain regarding the natural history of *M. genitalium*, its contribution to disease and long-term sequelae. A decline in cure rates for first-line anti-microbials has been observed. This is likely in part due to high usage of single-dose azithromycin in the sexual health field but also due to the intrinsic ability of *M. genitalium* to rapidly acquire anti-microbial resistance. Consequently, the term ‘the new STI superbug’ is not infrequently used by the media to describe this pathogen. Currently available antibiotics have side effects that, though rare, are potentially serious. This leads to inherent questions regarding the benefit of testing for and treating *M. genitalium*, particularly in asymptomatic individuals or in genital syndromes where the benefit of treatment is not well established. In this review, we summarize the most recent evidence and literature regarding *M. genitalium* and explore areas of research where disparities exist. We discuss the contribution of *M. genitalium* to genital syndromes, particularly those where data are conflicting, in order to inform indications for testing and treatment. Avoidance of increasing anti-microbial resistance with astute anti-microbial stewardship is paramount if we are to successfully manage *M. genitalium* infection. We examine the state of play regarding anti-microbial resistance and how to combat this, including currently available anti-microbials, resistance-guided therapy and novel therapeutic approaches. We aim to provide an overview of the current understanding of *M. genitalium* and the implications for current clinical practise and suggestions for future research.

## Introduction

*Mycoplasma genitalium* has been recognized as a sexually transmitted infection (STI) since it was first isolated from the urethra in two men with non-gonococcal urethritis (NGU) in the early 1980s^1^. It is an extremely slow-growing and fastidious organism to culture, and data regarding its association with genital syndromes accumulated more rapidly following development of the first polymerase chain reaction (PCR) test in the 1990s^2^. *M. genitalium* is now a well-recognized cause of acute and chronic NGU in men^1^. It is an established cause of cervicitis in women and emerging evidence supports its role as a causative agent of pelvic inflammatory disease (PID)^3^. However, its pathogenicity at other sites, including the rectum, remains subject to debate. Although the prevalence of *M. genitalium* infection is reported to be higher among persons living with HIV (PLHIV) than in the general population^4–6^, the interplay of *M. genitalium*–HIV co-infection is poorly understood. Anti-microbial resistance, including the emergence of multi-drug-resistant strains, is complicating current treatment strategies and there is a clear need for use of resistance assays and new classes of anti-microbials. In this review, we provide insights into our current understanding of *M. genitalium*’s clinical presentations in men and women and the interplay between *M. genitalium* and HIV infection. We explore implications of anti-microbial resistance for treatment strategies and novel therapeutic options.

## Recent developments regarding *M. genitalium*’s associations with disease outcomes: understanding the role of *M. genitalium* in women

Although there is a clear association of *M. genitalium* with cervicitis and emerging evidence supports an association with PID, further data regarding the pathogenic role of *M. genitalium* in the female genital tract are somewhat limited. Lis *et al*. found *M. genitalium* to be significantly associated with PID by meta-analysis (odds ratio of 2.14, 95% confidence interval [CI] 1.31–3.49)^3^, and *M. genitalium* was the sole pathogen identified by cervico-vaginal PCR in 5.5% of cases of PID from 2006 to 2017 at the Melbourne Sexual Health Centre^7^. Although PID is a clinical diagnosis that can be subjective, *M. genitalium* has been detected more frequently on endometrial biopsy in women with acute endometritis^8,9^ than in asymptomatic controls. Overall, the reported prevalence of PID in women with *M. genitalium* (4.8%) appears to be significantly less than in women with cervical *Chlamydia trachomatis* infection (18.6%, *P* = 0.006)^10^. Latimer *et al*. also reported that *M. genitalium* PID was associated with a lesser vaginal polymorphonuclear response compared with chlamydial PID^7^. These findings suggest that although *M. genitalium* can cause PID, it is a somewhat more indolent and less inflammatory pathogen than chlamydia. This has contributed to the uncertainty regarding the benefit of testing and treating of *M. genitalium* in PID, which is reflected in inconsistencies among international guidelines. The Centers for Disease Control and Prevention (US) guidelines do not routinely recommend *M. genitalium* testing for women with PID^11^, but it is recommended by guidelines of the British Association for Sexual Health and HIV (UK)^12^, the Australian Sexual Health Association (Australian)^13^ and the International Union against Sexually Transmitted Infections (European)^14^. Standard combination PID treatment in the majority of nations does not include an agent that is likely to cure *M. genitalium* infections. Hence, given the mounting evidence that supports a causal role in PID, the authors of this review believe data support testing for *M. genitalium* in women with PID, particularly where symptoms fail to respond rapidly to presumptive treatment. This applies particularly to high-risk settings, such as STI services where the prevalence of *M. genitalium* exceeds that in the general community. Baseline testing for *M. genitalium* in PID ensures prompt diagnosis and appropriate pathogen-specific treatment, which can reduce long-term sequelae of untreated PID such as tubal factor infertility and poorer pregnancy outcomes. Such reproductive health sequelae have been associated with *M. genitalium* by meta-analysis, although supporting data were more limited^3^. However, larger prospective studies of *M. genitalium* are required in women to inform public health implications and testing guidelines, particularly relating to natural history of infection and long-term sequelae of untreated infection.

## Contribution of *M. genitalium* to rectal and pharyngeal infection

Among asymptomatic men who have sex with men (MSM), *M. genitalium* appears to be detected significantly more often in the rectum than in the urethra^4,15–18^. At the urethral site, *M. genitalium* is an established cause of NGU and chronic NGU^1^. However, data are conflicting regarding the pathogenicity of *M. genitalium* at the rectum, its role in the development of rectal symptoms and as a causative agent of proctitis.

*M. genitalium* was found to be the sole pathogen in 12% of MSM with proctitis by Bissessor *et al*.^19^ and in 17% by Ong *et al*.^20^; the latter reported rates of *M. genitalium*–proctitis comparable to those of *C. trachomatis*–proctitis (21%)^20^. Bissessor *et al*. also reported that MSM with proctitis had significantly higher bacterial loads than asymptomatic controls and that MSM with rectal *M. genitalium* were significantly more likely to be HIV-positive^19^. Of note, these studies were among sexual health clinic attendees, whose prevalence of *M. genitalium* generally exceeds that of the general population. Conversely, several studies have reported rectal *M. genitalium* infection to be mostly asymptomatic^15,18,21^, and Read *et al*. found no significant difference in rates of rectal *M. genitalium* between MSM with proctitis and those with asymptomatic rectal infection^16^. Therefore, evidence suggests that rectal *M. genitalium* infection can result in asymptomatic carriage and less commonly proctitis. MSM with asymptomatic rectal infection may serve as reservoirs for onward transmission, whilst risk factors for the development of proctitis may involve factors such as individual host immune response, higher *M. genitalium* load or concurrent HIV. Overall, more data are needed to understand the role of host immunity and how this may impact on development of symptoms and duration of *M. genitalium* infection.

Rectal co-infection with bacterial STIs is common in MSM. A recent study at the Melbourne Sexual Health Centre found that 13 to 14% of rectal samples with *Neisseria gonorrhoea* and *C. trachomatis* had concurrent *M. genitalium* (1 in 6 cases)^17^ and that high rates of co-infection were likely to result in significant exposure of undetected *M. genitalium* to azithromycin. However, where *M. genitalium* is not contributing to symptomatology, the individual benefit of detection and treatment for *M. genitalium* remains unclear. Although screening for *M. genitalium* at the rectum is not currently recommended by international guidelines, the authors of this review believe that consideration should be given to testing for *M. genitalium* in men presenting with proctitis, particularly in high-risk settings such as sexual health centres or among MSM LHIV^14^. This does not necessarily need to be first-line but should be considered in men with proctitis who remain symptomatic after testing negative for other known pathogens. Data around pharyngeal infection are less heterogeneous; a recent meta-analysis^17^ provided a pooled estimate of 1% for *M. genitalium* at the pharynx in MSM^17,22^ and found no support for routine testing.

## Recent developments regarding the association of *M. genitalium* with HIV

*M. genitalium* is significantly more common in both women and MSM LHIV than in seronegative counterparts^6,7,23^. The majority of data are from retrospective studies of high-risk women, including sex workers in Africa, and limited data are from women and MSM LHIV in developed countries. For women in Africa, *M. genitalium* appears to be an independent risk factor for HIV acquisition, and two studies reported a twofold increased risk^6,24^. Currently, *M. genitalium* testing is not widely available in resource-poor settings, which bear a high burden of new HIV diagnoses. *M. genitalium* testing in female genital syndromes could be considered in resource-poor countries to reduce the burden from untreated chronic disease.

For women LHIV, chronic cervical *M. genitalium* infection has been associated with cervical secretion of pro-inflammatory infiltrates and enrichment of HIV target cells, theoretically increasing the risk of HIV transmission^25^. Studies among African women support this theory and have shown cervical HIV-RNA shedding to be associated with higher bacterial burdens^26^ or chronic *M. genitalium* infection^25^. However, a recent US study found no association^27^, suggesting that although cervical *M. genitalium* can increase risk of HIV transmission, other confounding factors, such as access to anti-retroviral therapy and serum HIV-RNA viral load, are at play.

Among MSM, it is plausible that rectal *M. genitalium* infection increases the risk of HIV transmission via increased rectal HIV-RNA shedding; however, there are no published data examining this association. Although Sadiq *et al*. found no association between NGU and increased urethral shedding of HIV-RNA^28^, *M. genitalium* was not examined specifically.

Overall, the immune-physiology of *M. genitalium*–HIV co-infection is poorly understood. The fastidious nature of *M. genitalium* enables it to establish chronic infection, which may assist in the evasion of host immune responses^29^. In addition, T-cell immunodeficiency associated with HIV may cause impaired or delayed spontaneous clearance of *M. genitalium*, which may explain the higher rates of *M. genitalium* infection we observe in PLHIV. Clinicians should maintain a lower threshold for *M. genitalium* testing among PLHIV, particularly those with symptoms of PID or proctitis.

## Current understanding of *M. genitalium* immune-physiology and the host immune response

Most research on the host response to *M. genitalium* is taken from studies regarding female genital tract infection. Limited data exist in men or for rectal infections. In the female reproductive tract, the first cells contacted by *M. genitalium* are vaginal and cervical epithelial cells (ECs). *M. genitalium* rapidly attaches to these ECs and establishes high intracellular titres within hours of infection^30^. Immunogenic *M. genitalium* proteins activate Toll-like receptors 2 and 6, which are expressed in high numbers within ECs^31^. This results in the production of inflammatory cytokines and chemokines, including interleukin 6 and 8 with subsequent, rapid leukocyte and macrophage recruitment^31^. Although *M. genitalium* is susceptible to such attack, it is hypothesized that the predominantly intracellular location infers a survival advantage, somewhat enabling evasion of these host cellular immune responses^30,31^.

*M. genitalium* serum IgA and IgG antibodies are detected among infected women significantly more than among uninfected controls^32^. The same finding has been demonstrated for *M. genitalium* IgG in men with NGU^33^. Of antigenic *M. genitalium* proteins, MgbP and MgpC are encoded by genes that exhibit extensive sequence diversity^32^. Interestingly, antibodies against MgbP and MgbC were predominantly detected on immunoblotting of vaginal and cervical samples in *M. genitalium*–infected women^32^. It is therefore plausible that genetic evolution of these antigens enables antibody evasion, increasing the likelihood of persistent infection.

## Recent advances in our understanding of anti-microbial resistance in *M. genitalium*

*M. genitalium* has intrinsically limited susceptibility to many commonly used anti-microbials because of its lack of a peptidoglycan-containing cell wall^34^. Therefore, treatment options are largely restricted to agents that target protein synthesis or DNA replication such as macrolides, tetracyclines, ketolides, streptogramins and extended-spectrum fluoroquinolones. Unfortunately, however, *M. genitalium* has shown a marked propensity to rapidly acquire resistance to available treatment options. Resistance to azithromycin, the most commonly used anti-microbial for *M. genitalium*, is due to mutations in the 23S ribosomal RNA molecule within the 50S subunit of the bacterial ribosome. These single-nucleotide polymorphisms (SNPs) in position 2058 and 2059 (*Escherichia coli* numbering) of the 23S ribosomal RNA gene result in high-level resistance to azithromycin. Macrolide resistance develops *de novo* in at least 12% of *M. genitalium* infections following single-dose 1 g azithromycin^35,36^. Some, but not all, data suggest that extended regimens of azithromycin may result in less selected resistance^35,36^. The widespread use of azithromycin in the STI field, particularly syndromically as a 1 g single dose, has led to a steep rise in macrolide resistance and marked decline in azithromycin cure over the past decade^37^. A recent meta-analysis has shown a rise in the global prevalence of macrolide resistance mutations from 10% before 2010 to 51% in 2017^38^, and the greatest increase was in the countries in the Western Pacific region (9–68%)^16,38^. Macrolide-resistant *M. genitalium* infections were also more common in MSM (69%) than in heterosexual men (40%)^39^, likely reflecting the higher use of azithromycin in this population to treat STIs.

Although the contribution of resistance mutations to failure of extended-spectrum fluoroquinolones has been harder to determine, data support an association between failure of moxifloxacin and sitafloxacin and a number of SNPs in the ParC region of the quinolone resistance-determining region, particularly at positions S83I and D87N. Concurrent GyrA mutations appear to increase the risk of failure of both agents^39^. Although moxifloxacin was highly effective for macrolide-resistant *M. genitalium* when first used in 2003^40^, treatment failures were first reported in 2008^41^, and meta-analysis has shown a decline in cure from 100% in studies prior to 2010 to 89% subsequently^42^. Although estimates of the global prevalence of fluoroquinolone resistance mutations (8%) are far lower than for macrolides, the highest resistance is again reported in countries in the Western Pacific region (14%)^38^. The emergence of dual-class-resistant strains poses considerable challenges to effective treatment and control of *M. genitalium*, and estimates are 3% globally but 7% in countries in the Western Pacific^38^.

## Strategies to improve treatment and control of *M. genitalium*: when to test and use of resistance assays

The prevalence of *M. genitalium* is similar to that of chlamydia, particularly in sexual health clinic attendees. However, significant complexities and concerns regarding treatment of asymptomatic infection mean that screening cannot be recommended at present. These include access to and cost of treatment, increasing anti-microbial resistance and associated drug toxicities^20,21^. Limited data exist regarding the natural history of *M. genitalium* and long-term sequelae of asymptomatic infection, and therefore priority lies with preserving currently available anti-microbials for the treatment of infections causing pathology and disease. The risk is that treatment of asymptomatic infection would cause iatrogenic complications without a clear benefit of microbiological cure. Until more efficacious drug regimens are available with fewer and less serious side effects, testing for *M. genitalium* should be limited to clinical scenarios supported by recent evidence. These include men with NGU, women with cervicitis or PID and MSM with proctitis (taking into consideration discussion points earlier in the article). Sexual contacts of confirmed cases, regardless of symptoms, should be offered testing and treatment, particularly where a sexual relationship is ongoing and risk of re-infection remains high. Wherever possible, nucleic acid amplification test/PCR assays, which enable simultaneous detection of macrolide resistance, should be used. This enables selection of antibiotics on the basis of evidence of macrolide resistance and reduces inappropriate antibiotic exposure and *de novo* resistance. Published evidence supports the benefits of macrolide resistance assays in clinical practice^43,44^, as detection of SNPs in the 23S ribosomal RNA gene of *M. genitalium* confers high-level resistance to azithromycin^45^. However, although detection of quinolone resistance mutations in the ParC gene with or without GyrA genes have been associated with failure of moxifloxacin and sitafloxacin^39^, cure has also been reported in the presence of these mutations, and the role of these assays in directing treatment strategies is less clear at present^46^.

## Anti-microbials on the horizon

The limited susceptibility and rapid acquisition of anti-microbial resistance have posed significant issues for treatment and control in *M. genitalium* and have led to investigation of a number of registered agents. Pristinamycin, a streptogramin that arrests protein synthesis, was evaluated in a case series against macrolide-resistant *M. genitalium* strains but achieved only 75% cure when used as either a 1 g dose four times daily or 1 g three times daily in combination with doxycycline 100 mg twice daily for 10 days^47,48^. With limited availability in many countries, its use has generally been restricted to cases of dual-class resistance or where fluoroquinolones are contraindicated, including pregnancy and breast-feeding. Minocycline, a tetracycline, appears to have more favourable mean inhibitory concentrations (MICs) than doxycycline for *M. genitalium* and has been reported to cure four patients in Japan and the US who had failed treatment with both a macrolide and a fluoroquinolone when used as an extended 14-day regimen (100 mg twice daily)^49,50^. Owing to its low cost and ease of availability, more data on this agent are needed, and a recent case series in Melbourne reported 71% (95% CI 54–85%) cure in 35 patients with macrolide-resistant *M. genitalium* who had failed moxifloxacin^48^. Spectinomycin, an aminocyclitol aminoglycoside, has been evaluated in a single patient who failed a number of anti-microbials and had a contraindication to fluoroquinolones. Based on favourable MIC data, it was used empirically in a 2 g intramuscularly daily dose for 7 days and achieved microbial cure^51^. Limited availability, high cost and administration via daily intramuscular injections create considerable barriers to its use and will impact greatly on further evaluation of this agent for *M. genitalium*.

Several new anti-microbials, including drugs from new classes, have emerged in the past decade. Although research and development is focused on infections with a high market yield, including community-acquired pneumonia, and priority STIs, such as *N. gonorrhoea*, their target profile has suggested activity against *M. genitalium*. The first of these was the fluoroketolide solithromycin. This was assessed in a limited number of patients with *M. genitalium* infections before concerns regarding hepatoxicity impacted on further evaluation. *In vitro* studies showed that solithromycin MICs were several dilutions lower than those of azithromycin for macrolide-resistant *M. genitalium* isolates, but some cross-resistance was evident^52^. Trials for solithromycin in *N. gonorrhoea* included a small number of *M. genitalium* co-infections, where a dose of 1200 mg cleared six out of seven *M. genitalium* infections but 1 g cleared only one out of three^53^. Lefamulin is a pleuromutilin that recently received approval from the US Food and Drug Administration for the treatment of community-acquired pneumonia. It binds to the 50S bacterial ribosome to inhibit protein synthesis, a mechanism of action that differs from that of macrolides, hence limiting cross-resistance. *In vitro* studies showed favourable MICs in macrolide-susceptible and -resistant *M. genitalium* strains and hence lefamulin is a promising agent for the treatment of *M. genitalium* and has additional activity against gonorrhoea^54^.

Lastly, a number of investigational fluoroquinolones, DNA gyrase and topoisomerase II inhibitors in the pipeline are entering trials for *N. gonorrhoea*. Zoliflodacin is a novel spiropyrimidinetrione and topoisomerase II inhibitor that inhibits DNA biosynthesis with a distinct mode of action that differs from that of other available anti-microbials. *In vitro* studies against 47 *M. genitalium* isolates, including moxifloxacin-resistant strains, revealed only one strain with increased MICs (4 mg/L) and potential resistance to zoliflodacin^55^. Overall, the authors considered zoliflodacin to be more potent than moxifloxacin and no cross-resistance was found between the two classes of topoisomerase II inhibitors. Zoliflodacin is active against both *N. gonorrhoeae* and *C. trachomatis* and therefore holds considerable appeal as a candidate for the syndromic management of STIs. Gepotidacin, a novel triazaacenaphthylene topoisomerase II inhibitor in trials against *N. gonorrhoea*, inhibits DNA replication via an alternate mechanism and target of action to fluoroquinolones, theoretically resulting in limited cross-resistance with other quinolones. *In vitro* studies demonstrated high activity against gonococcal strains and lower MICs than moxifloxacin in a limited number of *M. genitalium* isolates^56,57^, but more data are needed.

## Sequenced and combination strategies to improve cure

Doxycycline achieves cure rates of less than 30% in *M. genitalium* infections. However, studies have shown that the use of doxycycline for 1 week prior to resistance-guided therapy significantly lowers bacterial load and achieves higher cure rates than treatment with a macrolide or fluoroquinolone alone^43^. Cure rates in the order of 95% for sequential doxycycline-extended azithromycin and 92% for doxycycline-moxifloxacin were observed by Durukan *et al*.^44^. Sitafloxacin, a fourth-generation fluoroquinolone, has more favourable MICs than moxifloxacin and *in vitro* has been shown to cure some *M. genitalium* strains harbouring quinolone resistance mutations that reduce moxifloxacin susceptibility^2,44^. However, cure rates similar to those achieved with moxifloxacin (92%) were achieved using sequential doxycycline-sitafloxacin therapy in Melbourne for the treatment of macrolide-resistant *M. genitalium*^43^.

Combinations of anti-microbials have been used to optimize cure and minimize *de novo* resistance of bacterial infections with a propensity to develop anti-microbial resistance, such as tuberculosis and methicillin-resistant *Staphylococcus aureus*. The Melbourne Sexual Health Centre group recently trialled combination therapy with doxycycline (100 mg twice a day) and sitafloxacin (100 mg twice a day) for 7 days as a novel approach to treat highly resistant *M. genitalium* strains^57^. This regimen was well tolerated and cured 11 out of 12 infections that had failed prior treatment with sequenced doxycycline-moxifloxacin and doxycycline-pristinamycin^57^. Although sitafloxacin is more likely than moxifloxacin to cure an infection carrying an S83I (par C) mutation, concurrent gyrA mutations, particularly M95I, increase the risk of sitafloxacin failure. In this study, combination doxycycline+sitafloxacin cured *M. genitalium* strains with gyrA mutations, suggesting synergy between the two anti-microbials. Although this is promising, more data are needed to inform the use of combination doxycycline+sitafloxacin therapy to treat highly resistant *M. genitalium* strains.

## Conclusions

*M. genitalium* continues to pose complex clinical diagnostic and treatment challenges. Much remains unknown regarding its pathogenic role and the host immune response which appears to vary between both individuals and sites of infection. In this manner, it has proven itself to be rather different from other bacterial STIs such as *C. trachomatis* and *N. gonorrhoea*. Further evidence is required to fully understand its role in female reproductive health sequelae and proctitis. A more detailed understanding of the natural history of asymptomatic, untreated infection is needed to inform screening guidelines, particularly in young women where infection could result in adverse reproductive health outcomes. With anti-microbial resistance increasing expeditiously, it is possible that *M. genitalium* will become the first ‘untreatable’ STI. Judicial anti-microbial prescribing in all health-care settings with specific measures to reduce *de novo* resistance in *M. genitalium* strains is imperative. This calls for widespread clinician education in sexual health and primary care specialities regarding the use of our most effective first-line strategies. Although new drugs show promise, many will not be available for some time and may prove costly and difficult to access, particularly for primary health-care providers. Early data indicate that combination therapy with sitafloxacin and doxycycline is well tolerated and effective for highly resistant *M. genitalium* strains, but access to sitafloxacin is limited in many parts of the world. In the meantime, efforts should be directed to epidemiological surveillance to quantify the burden of *M. genitalium* infection and determine trends in resistance by population and geographical area. This is essential to further inform the development and revision of local and national treatment guidelines and strategies.
